# On the van der Waals Gas, Contact Geometry and the Toda Chain

**DOI:** 10.3390/e20080554

**Published:** 2018-07-26

**Authors:** Diego Alarcón, P. Fernández de Córdoba, J. M. Isidro, Carlos Orea

**Affiliations:** Instituto Universitario de Matemática Pura y Aplicada, Universidad Politécnica de Valencia, 46022 Valencia, Spain

**Keywords:** van der Waals gas, contact geometry, Toda chain

## Abstract

A Toda–chain symmetry is shown to underlie the van der Waals gas and its close cousin, the ideal gas. Links to contact geometry are explored.

## 1. Introduction

The contact geometry of the classical van der Waals gas [1] is described geometrically using a five-dimensional contact manifold M [2] that can be endowed with the local coordinates *U* (internal energy), *S* (entropy), *V* (volume), *T* (temperature) and *p* (pressure). This description corresponds to a choice of the fundamental equation, in the energy representation, in which *U* depends on the two extensive variables *S* and *V*. One defines the corresponding momenta T=∂U/∂S and −p=∂U/∂V. Then, the standard contact form on M reads [3,4]

(1) α=dU+TdS−pdV.

One can introduce Poisson brackets on the four-dimensional Poisson manifold P (a submanifold of M) spanned by the coordinates *S*, *V* and their conjugate variables *T*, −p, the nonvanishing brackets being

(2) {S,T}=1,{V,−p}=1.

Given now an equation of state

(3) f(p,T,…)=0,

one can make the replacements T=∂U/∂S, −p=∂U/∂V in order to obtain

(4) $$f\left(-\frac{\partial U}{\partial V},\frac{\partial U}{\partial S},\ldots \right)=0.$$

In Ref. [5], we have called Equation (4) a partial differential equation of state (PDE of state for short). It plays a role analogous to that played by the Hamilton–Jacobi equation in classical mechanics [2,6,7]. With respect to the latter, however, there is one fundamental difference. While in mechanics the Hamilton–Jacobi equation is just one equation (regardless of the number of degrees of freedom), in thermodynamics, we have one PDE of state per degree of freedom because the defining equation of each momentum qualifies as an equation of state.

## 2. The PDEs of State of the van der Waals Gas

Let us consider one mole of particles of van der Waals gas (i.e., Avogadro’s number *N* of particles). The fundamental equation in the energy representation U=U(S,V) reads [1]

(5) $$U\left(S,V\right)=U_{0}{\left(\frac{V_{0}}{V-b}\right)}^{2/3}\exp\left(\frac{2S}{3Nk_{B}}\right)-\frac{a}{V},$$

with $U_{0},V_{0}$ certain fiducial values; setting a=0 and b=0, one recovers the ideal gas. The variables *T* and −p, conjugate to *S* and *V*, are

(6) $$T=\frac{\partial U}{\partial S}=U_{0}{\left(\frac{V_{0}}{V-b}\right)}^{2/3}\exp\left(\frac{2S}{3Nk_{B}}\right)\frac{2}{3Nk_{B}}$$

and

(7) $$p=-\frac{\partial U}{\partial V}=\frac{2}{3}U_{0}\exp\left(\frac{2S}{3Nk_{B}}\right)\frac{V_{0}^{2/3}}{{\left(V-b\right)}^{5/3}}-\frac{a}{V^{2}}.$$

Equations (6) and (7) lead to the van der Waals equation of state

(8) $$\left(p+\frac{a}{V^{2}}\right)\left(V-b\right)=Nk_{B}T$$

and the equipartition theorem:

(9) $$U\left(T,V\right)=\frac{3}{2}Nk_{B}T-\frac{a}{V}.$$

The first PDE of state follows from Equation (8),

(10) $$\left(\frac{\partial U}{\partial V}-\frac{a}{V^{2}}\right)\left(V-b\right)+Nk_{B}\frac{\partial U}{\partial S}=0,$$

while, from Equation (9), we obtain the second PDE of state:

(11) $$U-\frac{3}{2}Nk_{B}\frac{\partial U}{\partial S}+\frac{a}{V}=0.$$

when a=0 and b=0, systems (10) and (11) correctly reduce to the corresponding system of PDEs for the ideal gas, obtained in Ref. [5]. One readily verifies that integration of the systems (10) and (11) lead back to the fundamental Equation (5) we started off with.

## 3. Relation to the Toda Chain

Although well studied in the literature [8,9,10], for the benefit of the reader, we very briefly summarise the essentials of Toda lattices needed for our purposes here. The Toda chain is a model for a nonharmonic lattice describing the motion of a chain of particles subject to nearest-neighbour interactions. The statement that interactions are restricted to nearest neighbours translates into an equation of motion for the *n*–th particle

(12) $$m_{n}{\ddot{x}}_{n}\left(t\right)=\nabla V\left(x_{n+1}\left(t\right)-x_{n}\left(t\right)\right)-\nabla V\left(x_{n}\left(t\right)-x_{n-1}\left(t\right)\right),$$

where $x_{n}\left(t\right)$ is its displacement from equilibrium, and *V* is a certain potential function. Toda assumes the latter to be given by the exponential of the relative displacements:

(13) $$V=\exp\left(-(x_{n}-x_{n-1})\right).$$

Although the resulting model turns out to exhibit many interesting properties, integrability being one of them, the succinct summary just given is all we will need for our purposes.

Returning now to our problem, a succession of changes of variables in configuration space C (the submanifold of M spanned by the extensive coordinates S,V) will relate the fundamental Equation (5) for the van der Waals gas to the potential energy of the Toda chain. We define the new variables $S^{\prime },V^{\prime }$

(14) $$S^{\prime }:=S,\;V^{\prime }:=V-b,$$

and s,v

(15) $$s:=\frac{S^{\prime }}{Nk_{B}},\;v:=\ln\left(\frac{V^{\prime }}{V_{0}}\right),$$

in terms of which the fundamental Equation (5) reads

(16) $$U\left(s,v\right)=U_{0}\exp\left[\frac{2(s-v)}{3}\right]-\frac{a}{V_{0}e^{v}+b}.$$

The transformations (14) and (15) are both diffeomorphisms: they can be inverted, regardless of the values of the van der Waals parameters a,b. However, the final change of variables

(17) $$x:=s-v,\;U_{0}\exp\left(\frac{2y}{3}\right):=\frac{a}{V_{0}e^{v}+b}$$

becomes singular when a=0. For the moment, we proceed under the assumption that a≠0, so Equation (17) is invertible. Then, the fundamental Equation (16) becomes

(18) $$U\left(x,y\right)=U_{0}\left[\exp\left(\frac{2x}{3}\right)-\exp\left(\frac{2y}{3}\right)\right]=W\left(x\right)-W\left(y\right),$$

where we have defined the new function

(19) $$W\left(z\right):=U_{0}\exp\left(\frac{2z}{3}\right).$$

The function W(z) coincides with the potential function of the Toda chain; we have already encountered it in Ref. [5] in the context of the ideal gas. Since the latter has a=0, which causes the change of variables (17) to be singular, one must proceed differently in this case. Instead of Equation (17), a nonsingular change of variables to consider for the ideal gas is

(20) $$x^{\prime }:=s-v,\;y^{\prime }:=s+v.$$

As already seen in Ref. [5], this yields a fundamental equation depending on $x^{\prime }$, but not on $y^{\prime }$:

(21) $$U_{\mathrm{ideal}}\left(x^{\prime }\right)=W\left(x^{\prime }\right).$$

On the other hand, from Ref. [8], we know that, in the limit of small wave amplitudes, the time average of the momentum variable in a thermal ensemble of Toda chains is directly proportional to the product of Boltzmann’s constant $k_{B}$ times the temperature *T* (see Equation (3.20) of Ref. [8], the right-hand side of which is independent of the lattice site *n*). We conclude that, *in the limit of small amplitudes, a thermal ensemble of waves in the Toda chain behaves exactly as an ideal gas*.

Returning now to the van der Waals gas in Equation (18), the new canonical momenta read

(22) $$p_{x}=\frac{\partial U}{\partial x}=\frac{2}{3}W\left(x\right),\;p_{y}=\frac{\partial U}{\partial y}=-\frac{2}{3}W\left(y\right).$$

While the momentum $p_{x}$ is the same as for the ideal gas, the negative sign in $p_{y}$ can be traced back to the reduction in energy, with respect to the ideal case, due to the van der Waals parameter *a*. The PDEs of state read, in the new variables x,y,

(23) $$\frac{\partial U}{\partial x}-\frac{2U_{0}}{3}\exp\left(\frac{2x}{3}\right)=0,\;\frac{\partial U}{\partial y}+\frac{2U_{0}}{3}\exp\left(\frac{2y}{3}\right)=0.$$

Compared to Equations (10) and (11), we see that, in the new variables x,y, the PDEs of state decouple into a system of two identical equations (up to a sign), one for each independent variable. Moreover, the equation corresponding to the variable *x* equals that PDE of the ideal gas, which expresses the equipartition theorem. Finally the contact form (1) reads, in terms of x,y and the corresponding momenta $p_{x},p_{y}$,

(24) $$\alpha =dU+p_{x}dx+p_{y}dy.$$

In the limit when the gas is ideal, the momentum $p_{y}$ vanishes identically [5], and the physics is described in terms of the three-dimensional contact submanifold N spanned by $x,p_{x}$ and *U*.

## 4. Discussion

The physics of the classical van der Waals gas is usually described by a five-dimensional contact manifold M endowed with the contact form given in Equation (1). In this paper, we have identified one particular diffeomorphism that neatly disentangles the (rather abstruse) fundamental Equation (5) to the much more manageable form given by Equations (18) and (19). This latter form is not just easier to work with; it is also more inspiring. Namely, the fundamental equation of the van der Waals gas now equals the difference of two terms (one term per independent variable x,y), each one of which is a copy of the Toda potential function [8,9,10].

From the point of view of contact geometry, the only difference between the van der Waals gas and the ideal gas lies in the fact that the contact manifold describing the van der Waals gas remains five-dimensional, instead of reducing to the three-dimensional contact submanifold N we found in the ideal case [5]. However, as we have proved in Equation (18), the fundamental equation can be expressed in terms of the Toda potential function in both cases.

Why the precisely Toda potential should arise in this thermodynamical context, instead of some other potential function, is a question that arises naturally. We believe the answer is the following. The distinguishing feature of the Toda potential is the exponential function. In thermodynamics, the exponential function arises naturally through Boltzmann’s principle: the number of microstates that are compatible with a given macrostate specified by the value *S* of the entropy is proportional to $\exp(S/k_{B})$. That the latter factor is present in the fundamental Equation (5) should come as no surprise, since the internal energy should be an extensive variable of the system.

Another intriguing feature of the above correspondence between the fundamental equation of a gas (either ideal or van der Waals) and the Toda potential function is the following. The small–amplitude limit considered in Ref. [8] is the limit of vanishing kinetic energy; this fact is reflected in the vanishing (to first order of approximation) of the time average of the generalised velocities ${\dot{s}}_{n}$ in Ref. [8]. This limit has been called *the topological limit* in Ref. [11]; roughly speaking, it amounts to cancelling the kinetic term while keeping only the potential term in the Hamiltonian. This fact allows us to sharpen our previous correspondence, which we can now state more precisely as follows: *the classical thermodynamics of the (ideal or van der Waals) gas has a dual theory which, to first order of approximation, coincides with the topological limit of a thermal ensemble of waves in the Toda chain*. Surprising here is the fact that, for the ideal gas, all energy is purely kinetic, and the potential energies introduced by the van der Waals parameters a,b are almost negligible compared to the kinetic energy. Thus, *the theory of gases, where energies are completely or mostly kinetic, is mapped by this correspondence into a dual theory in which kinetic energies are negligible*. Vanishing or at least negligible kinetic energies are strongly reminiscent of topological field theory [12]; we hope to report on this issue in the future, as well as on its relation to Riemannian fluctuation theory [13,14].
